# Interplay between VEGF and Nrf2 regulates angiogenesis due to intracranial venous hypertension

**DOI:** 10.1038/srep37338

**Published:** 2016-11-21

**Authors:** Liwen Li, Hao Pan, Handong Wang, Xiang Li, Xiaomin Bu, Qiang Wang, Yongyue Gao, Guodao Wen, Yali Zhou, Zixiang Cong, Youqing Yang, Chao Tang, Zhengwei Liu

**Affiliations:** 1 Department of Neurosurgery, Jinling Hospital, School of Medicine, Nanjing University, Nanjing, China; 2 Department of Clinical Laboratory, Jinling Hospital, School of Medicine, Nanjing University, Nanjing, China

## Abstract

Venous hypertension(VH) plays an important role in the pathogenesis of cerebral arteriovenous malformations (AVMs) and is closely associated with the HIF-1α/VEGF signaling pathway. Nuclear factor erythroid 2-related factor 2(Nrf2) significantly influences angiogenesis; however, the interplay between Nrf2 and VEGF under VH in brain AVMs remains unclear. Therefore, our study aimed to investigate the interplay between Nrf2 and VEGF due to VH in brain AVMs. Immunohistochemistry indicated that Nrf2 and VEGF were highly expressed in human brain AVM tissues. *In vivo*, we established a VH model in both wild-type (WT) and siRNA-mediated Nrf2 knockdown rats. VH significantly increased the expression of Nrf2 and VEGF. Loss of Nrf2 markedly inhibited the upregulation of VEGF, as determined by Western blot analysis and qRT-PCR. *In vitro*, primary brain microvascular endothelial cells (BMECs) were isolated from WT and Nrf2^−/−^ mice, and a VEGF-Nrf2 positive feed-back loop was observed in BMECs. By trans well assay and angiogenesis assay, Nrf2 knockout significantly inhibited the migration and vascular tube formation of BMECs. These findings suggest that the interplay between Nrf2 and VEGF can contribute to VH-induced angiogenesis in brain AVMs pathogenesis.

Intracranial arteriovenous malformations (AVM) can cause hemorrhagic stroke in young and children^1^. AVM is a congenital vascular lesion arising from a disruption of normal vascular morphogenesis during fetal development, consisting of a complex and tangled network of abnormal arteries and veins without an intervening capillary bed, along with abnormal circumfluent veins shown on angiography^2^,^3^. However, the etiology of AVMs has yet to be mechanistically determined. In 1997, Lawton *et al*.^4^ first proposed an angiogenesis hypothesis for AVM formation and found significant relationships between venous hypertension and angiogenic activity. Further investigation demonstrated that venous hypertension was an important and primary event in the pathogenesis of AVMs^5^,^6^ and was closely associated with the upregulation of vascular endothelial growth factor (VEGF) and hypoxia-inducible factor 1-alpha(HIF-1α)^7^.

Nrf2 is a pivotal transcription factor that regulates various antioxidant genes such as heme oxygenase-1 (HO-1) and NAD(P)H: quinine oxidoreductase-1(NQO1). Under physiological conditions, Nrf2 is sequestered in the cytoplasm by a Kelch-like ECH associated protein 1 (Keap1) and remains inactive. However, upon exposure to oxidative stress, Nrf2 is released from Keap1, translocates into the nucleus, and stimulates antioxidant response element (ARE)-dependent phase II gene expression^8^,^9^,^10^,^11^. Preliminary studies in our laboratory explored the protection of Nrf2 in traumatic brain injury^12^,^13^,^14^,^15^,^16^. An increasing number of studies supporting the pivotal role of Nrf2 in angiogenesis show that Nrf2 may promote vascular development via protection of retina from hyperoxia-induced oxidative stress^17^; these studies also indicate that Nrf2 blockade suppresses tumor cell angiogenesis and migration *in vivo* and *in vitro*^18^,^19^,^20^. These results suggest that Nrf2 can regulate angiogenesis via HO-1 mediated HIF-1α/VEGF signaling pathways.

Interestingly, Kweider *et al*. were the first to suggest that VEGF can activate Nrf2 in human choriocarcinoma BeWo cells and that this effect can be completely canceled by Nrf2-specific shRNA^21^. Kweider *et al*. finally hypothesized that VEGF might upregulates VEGF via Nrf2/HO-1/CO pathways to promote abnormal angiogenesis. However, whether the existence of the VEGF-Nrf2 loop in cerebral AVM has yet to be verified. In addition, both venous hypertension and transcription factor Nrf2 might regulate angiogenesis via upregulation of VEGF and HIF-1α. However, the relationship between venous hypertension and Nrf2 in the pathogenesis of AVMs remains unknown.

On the basis of previous studies, we propose that the interplay between VEGF and Nrf2 regulates angiogenesis under venous hypertension in AVMs. These findings may elucidate the pathogenic mechanism of AVMs at the molecular level and partly explain the etiology and development of AVMs.

## Results

### Expression of Nrf2 and VEGF in AVM tissues and its correlation with MVD values

**Figure 1:**
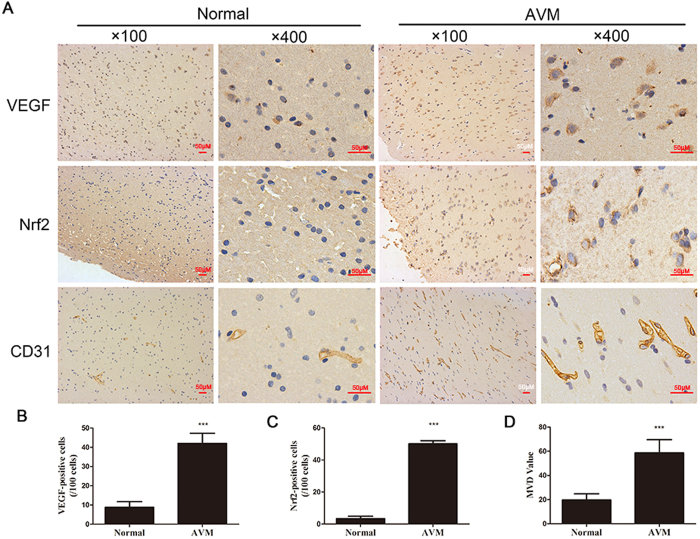
High expression of VEGF and Nrf2 along with MVD values in human AVMs tissues. Photomicrographs showing immunohistochemical staining for VEGF, Nrf2 and CD31 was shown. (**A**) Brown color spots were positively stained cells. Quantification analysis of immunoreactive cells for Nrf2, VEGF and CD31. (**B–D**) Data are presented as mean ± S.D. ***p < 0.001 compared with the normal group.

The expression levels of Nrf2 and VEGF were detected by immunohistochemistry in human AVM samples and normal brain tissues. As shown in Fig. 1, only a small number of Nrf2 and VEGF stained cells were observed in normal tissues with low MVD values, whereas a significantly large number of Nrf2 and VEGF stained cells were observed in AVM tissues with high MVD values. Immunoreactivity of Nrf2 and VEGF was detected mainly in the cytoplasm combining with some positive labels in the nuclei.

### Venous hypertension strongly activates Nrf2-ARE and HIF-1α/VEGF signaling pathways

**Figure 2:**
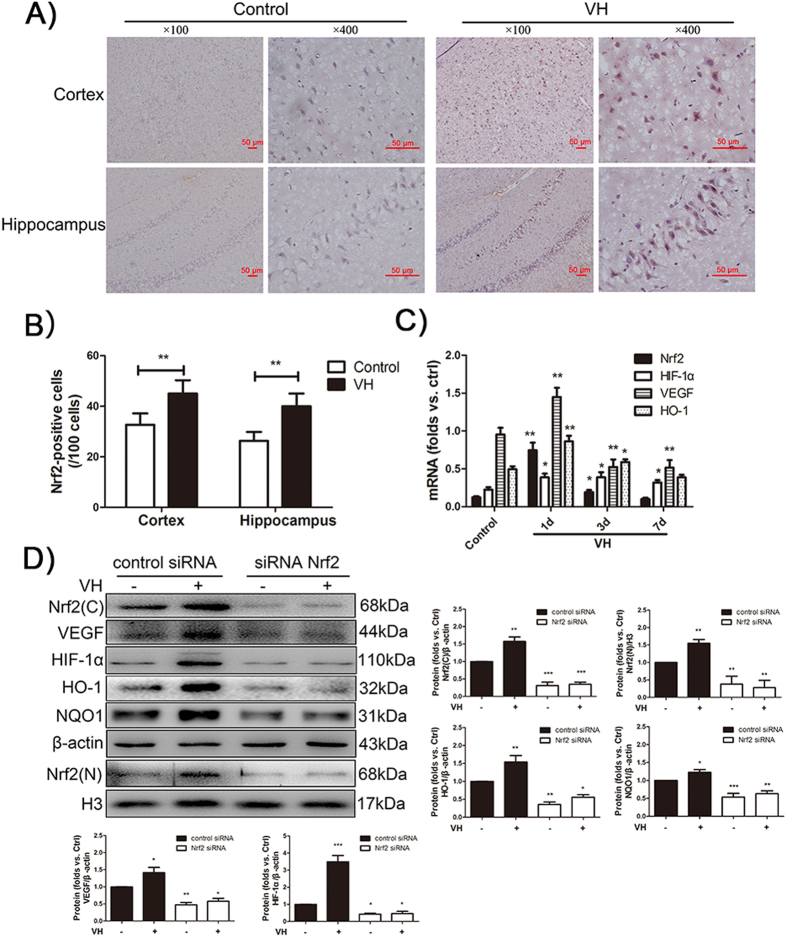
VH activates Nrf2-ARE and HIF-1α/VEGF signaling pathways. (**A**) Photomicrographs showing immunohistochemical staining for Nrf2 in rat brain tissues. Brown color spots were positively stained cells. (**B**) The number of immunostained Nrf2 cells was calculated in VH group compared to control group on Day 1 in both the cortex and the hippocampus. **P < 0.001 compared to control group. (**C**) qRT-PCR analysis showed the mRNA level of Nrf2, HIF-1α, VEGF and HO-1 at day 1, day 3 and day 7 after administration of CCA–EJV anastomosis. Data are presented as mean ± S.D. *p < 0.05, **p < 0.01, ***p < 0.001 versus control group. (**D**) Expression of VEGF, Nrf2 and its downstream target proteins such as HO-1, NQO1, HIF-1α, was examined by Western blot at day 1 after administration of CCA–EJV anastomosis in Nrf2-transfected rats. All images of western blot were representative images from three independent experiments and β-actin was used as a loading control. *P < *0.05*, **P < *0.01*, ***P < *0.001*. *Different from control si-RNA.

Immunohistochemical staining for Nrf2 was performed in the cortex and hippocampus of all rats. As shown in Fig. 2A, there were no or only faint expression of Nrf2 in both the cortex and the hippocampus of the control group. Meanwhile, Nrf2 was strongly expressed in the cortex and hippocampus of the VH group on Day 1 after CCA–EJV anastomosis. Digital image analysis indicated that the number of immunostained Nrf2 cells was significantly larger in the VH group than in the control group on Day 1 (cortex: 45 ± 5 vs. 33 ± 5 cells; hippocampus: 40 ± 5 vs. 26 ± 4 cells).

The time course of activation of the Nrf2-ARE and HIF-1α/VEGF signaling pathways induced by VH was further investigated. The expression levels of Nrf2, HIF-1α, VEGF, and HO-1 on Days 1, 3, and 7 after CCA–EJV anastomosis were evaluated using qRT-PCR. As shown in Fig. 2B, the mRNA expression of Nrf2 significantly increased relative to that of the control group after Day 1 of anastomosis and then began to decrease on Day 3. Similarly, the Nrf2 downstream target genes—i.e., HIF-1α, VEGF, and HO-1— also markedly increased on Day 1after the performence of anastomosis; this occurrence coincided with Nrf2 up regulation.

### Nrf2 knockdown inhibits VH induced-activation of Nrf2-ARE and HIF-1α/VEGF signaling pathway

Nrf2 is a critical regulating factor of intracellular antioxidants and phase II detoxification enzymes as an adaptive response to outside oxidative stress stimulus. To clarify the role of Nrf2 in response to VH, short interfering RNA transfection *in vivo* was used in our study. First, siRNA–Nrf2 or siRNA-control was transfected into the rat brain. After 24 h of transfection, Nrf2 expression was detected by Western blot analysis. Compared with the siRNA control, transfection with siRNA–Nrf2 significantly abated Nrf2 protein expression (p < 0.05, Fig. 2C). These results indicated that transfection with siRNA–Nrf2 was effective in rat brain.

Subsequently, transfection with siRNA–Nrf2 significantly eliminated Nrf2 up regulation induced by VH, as shown in Fig. 2C. Upregultion of VEGF, HIF-1α, and Nrf2 downstream target proteins such as HO-1 and NQO1 was also inhibited by Nrf2 knockdown (Fig. 2C). These results demonstrated that Nrf2 plays a critical role in the activation of the Nrf2-ARE and HIF-1α/VEGF signaling pathways induced by VH in rat brain.

### VEGF_165_ activates Nrf2-ARE via ERK1/2 pathway

**Figure 3:**
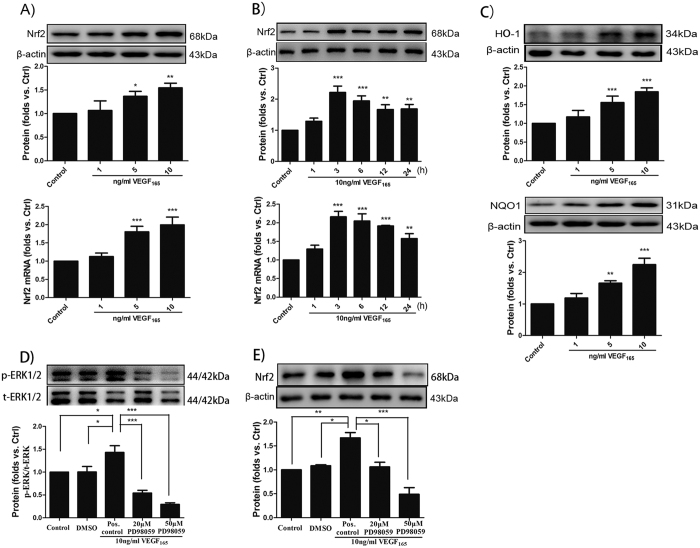
VEGF_165_ activates Nrf2-ARE signaling pathways via ERK/12 pathways. (**A**) Western blot and qRT-PCR were used to measure the expression of Nrf2 in response to various concentrations of VEGF_165_ stimulation in mice BMECs for 6 h. (**B**) 10 ng/ml of VEGF_165_ was used to stimulate BMECs at different time points, and the expression of Nrf2 was detected by Western blot and qRT-PCR. The mean of three independent experiments is shown. Data are presented as -fold induction after treatment compared with untreated cells (control = 1). *p < 0.05; **p < 0.01; ***p < 0.001; versus the control. (**C**) Nrf2 downstream target factors HO-1 and NQO1 were also detected by Western blot. (**D,E**) Following pretreated with MEK1/2 inhibitor PD98059 (20 and 50 μM) for 30 min, 10 ng/ml of VEGF_165_ was added to the BMECs for 3 h. The densities of corresponding phospho-ERK1/2, ERK1/2 and Nrf2 bands were measured, and the ratio was calculated. The mean of three independent experiments is shown. Pos., positive. *p < 0.05; **p < 0.01; ***p < 0.001; versus the control (one representative Western blot is shown; n = 3).

To investigate the efficacy of VEGF_165_ in activating the Nrf2-ARE signaling pathway, Western blot analysis and qRT–PCR were performed. First, BMECs were underwent serum deprivation (0.5% FBS), and no ECGs were present for 24 h before treatment with VEGF_165_. Nrf2 expression was then determined in a dose-response assay at concentrations of up to 10 ng/ml VEGF_165_ in primary murine BMECs after 6 h of incubation. As presented in Fig. 3A, the expression of Nrf2 was significantly elevated compared with the control group in a dose-dependent manner at 6 h. Subsequently, 10 ng/ml of VEGF_165_ was administered to BMECs at different time points to explore the time dependence of the activation of Nrf2-ARE in response to VEGF_165_. As early as 1 h after VEGF_165_ was administere, Nrf2 increased and remained significantly high for at least 12 h (Fig. 3B). Moreover, the protein expression of Nrf2 target genes, i.e. HO-1 and NQO1, was elevated 3 h after VEGF_165_ was administered (Fig. 3C). To further address the role of MAPK pathways in regulating Nrf2 by VEGF_165_, BMECs were pretreated with varying concentrations of the MEK1/2 inhibitor PD98059 for 30 min. Treatment with VEGF_165_ (10 ng/ml) for 3 h followed. Cell extracts were analyzed for Nrf2, total ERK1/2 and phosphorylated ERK1/2 by Western blot analyss. VEGF_165_ activated ERK1/2; 20 μM and 50 μM of PD98059 inhibited ERK1/2 activation compared with the VEGF_165_-treated cells (Fig. 3D), which were supposed to be a positive control. The results clearly indicated that the activation of ERK1/2 was a precondition for Nrf2 upregulation by VEGF_165_.

### Nrf2 up-regulates VEGF via Nrf2/HO-1/HIF-1α pathways

**Figure 4:**
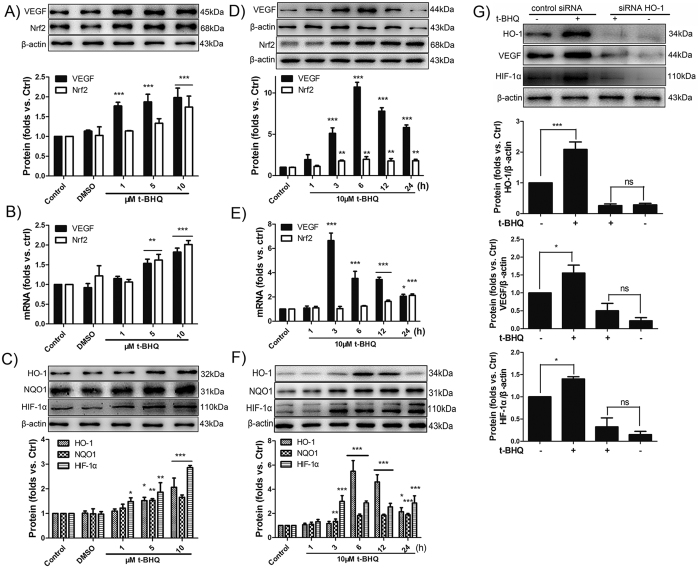
Nrf2 activator t-BHQ activates VEGF via Nrf2/HO-1/HIF-1α pathways. (**A,B**) After the BMECs was incubated with 1–10 μM of t-BHQ for 6 h, the mRNA and protein levels of Nrf2 and VEGF was determined using β-actin as the loading control by Western blot and qRT-PCR. The histograms show the ratio of Nrf2/β-actin and VEGF/β-actin. (**C**) Western blot shows the upregulation of Nrf2 downstream protein HO-1, NQO1 and HIF-1α in response to t-BHQ. (**D,E**) 10 μM t-BHQ was used to stimulate the BMECs at different time points, and the protein and mRNA levels of VEGF and Nrf2 were measured. (**F**) The expression of HO-1, NQO1, and HIF-1α were also measured and calculated. (**E**) After 48 h of transfection with siRNA–HO-1 or control siRNA, the BMECs were incubated with 10 μM t-BHQ for 6 h. Then, Western blot was used to analyse the expression of HO-1, VEGF and HIF-1α. *p < 0.05; **p < 0.05; ***p < 0.001 versus the control siRNA.

To explore whether Nrf2 activators upregulate the expression of VEGF in our system, BMECs were stimulated with 1–10 μM tertiary butylhydroquinone (t-BHQ) for 6 h. The expression levels of Nrf2-ARE and VEGF/HIF-1α were evaluated by Western blot analysis and qRT–PCR. We observed that t-BHQ increased the protein and mRNA levels of Nrf2 and VEGF at concentration ranging from 1 μM to 10 μM (Fig. 4A). The expression of downstream factors such as HO1, NQO1 and HIF-1α were alaso upregulated (Fig. 4B). BMECs were then treated with 10 μM of t-BHQ at varying time points to examine the time dependence of VEGF expression in response to t-BHQ. As early as 3 h following the administration of t-BHQ, VEGF increased, reaching the peak value at 6 h and remaining elevated for at least 24 h (Fig. 4C). At the mRNA level, the results of qRT–PCR revealed that the peak value of VEGF was achieved at 3 h, earlier than the peak of protein expression. Meanwhile, the mRNA and protein levels of Nrf2 were gradiently elevated from 3 h and remained high for up to 24 h. Similarly, 3 h after t-BHQ was administered, the related protein and mRNA expression levels of HO-1, NQO1, and HIF-1α were elevated relative to those of the control group (Fig. 4D).

To elucidate whether the upregulation of VEGF induced by t-BHQ occurred via the Nrf2/HO-1/HIF-1α pathways, we transfected BMECs with HO-1 siRNA. We subsequently analyzed the expression of HIF-1α and VEGF in response to t-BHQ. After 48 h of transfection, the protein expression of Nrf2 was detected by Western blot analysis. Compared with siRNA control, transfection with siRNA–HO-1 significantly knocked down the protein expression of HO-1 (p < 0.05, Fig. 4E). These results indicated that transfection with siRNA–HO-1 was effective in BMECs.

Transfection with siRNA–HO-1 significantly inhibited the upregulation of VEGF and HIF-1α by t-BHQ (p < 0.05) (Fig. 4F). The results suggested that the Nrf2 activator t-BHQ up-regulates VEGF in a time- and concentration-dependent manner via the Nrf2/HO-1/HIF-1α pathways.

### Nrf2 knockout suppresses the migration and tube formation of BMECs induced by VEGF_165_

**Figure 5:**
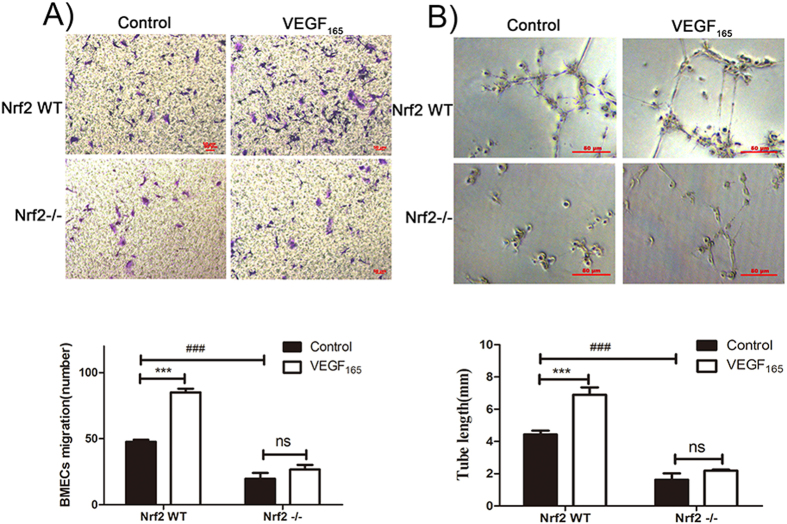
Nrf2 knockout attenuates VEGF_165_-induced migration and tube formation of BMECs. (**A**) BMECs migration with or without VEGF_165_ (100 ng/mL)was measured using a trans-well (100× magnifications). (**B**) Three dimensional Matrigel assay was performed to investigate the effect of Nrf2 knockout in tube formation of BMECs with or without VEGF_165_ (100 ng/mL). (400× magnifications) Data are expressed as the mean ± SD of at least three independent experiments. *p < 0.05; **p < 0.01; ***p < 0.001.

The migration of BMECs plays a crucial role in angiogenesis. To evaluate the effect of Nrf2 knockout on the migration of BMECs induced by VEGF_165_, trans-well assay was used. As shown in Fig. 5A, the number of migration cells of Nrf2−/− BMECs was markedly lower than that of Nrf2 WT BMECs. These results indicated that Nrf2 knockout significantly inhibited the migration of BMECs. In addition, in the Nrf2 WT group, the number of cells treated with 10 ng/ml VEGF_165_ that migrated to the filters in the migration assay were significantly higher than the VEGF_165_-free group (***P < 0.001). However, in the Nrf2−/− group, the effect of VEGF_165_ was significantly inhibited, suggesting Nrf2 significantly affects angiogenesis induced by VEGF_165_.

To verify the pivotal role of Nrf2 in the angiogenesis of BMECs, we performed the tube formation assay. BMECs of Nrf2 WT and Nrf2−/− were incubated with supplement-free DMEM/F12 medium with or without 10 ng/ml of VEGF_165_. The tube length of Nrf2 WT BMECs incubated with VEGF_165_ was markedly higher than that of the VEGF_165_-free group (***P < 0.001) (Fig. 5C). However, in the Nrf2−/− group, no significant difference in tube length was indicated between the VEGF_165_ group and the VEGF_165_-free group, which further supports the hypothesis that Nrf2 participates in the migration and tube formation of BMECs induced by VEGF_165_.

## Discussion

Substantial studies confirm that Nrf2 is a cytoprotective factor in regulating a battery of antioxidants and cellular protective genes, primarily in response to oxidative stress. The pro-angiogenesis effect of Nrf2 has recently been extensively investigated. Other studies demonstrated that the expression of Nrf2 is increased and activated during vascular developmentand that genetic ablation of Nrf2 reduced vascular density as well as endothelial cells sprouting^22^. Meanwhile, Nrf2 knockdown inhibited the OxPL-induced elevation of VEGF mRNA and endothelial cell sprout formation and migration^23^,^24^. Kuang *et al*. and Meng *et al*. found that hypoxia or arsenic can upregulate the mRNA and protein expression of Nrf2, heme oxygenase-1(HO-1), and VEGF, with a concomitant increase in cell migration and vascular tube formation; Nrf2 knockdown markedly decreased HO-1 and VEGF expression^25^,^26^. Further oncological studies also indicate that Nrf2 palys an important role in regulating angiogenesis in various types of cancer and that Nrf2 knockdown in human colon cancer cells significantly inhibit tumor growth in mouse xenograft^20^,^27^. Hypoxia due to rapid tumor growth can activate the Nrf2-ARE pathways, promoting the proliferation tumor pathological vessels. The absence of Nrf2 can lead to a decline in capillary density of tumor tissues by imposing a blockade to HIF-1α signaling. Similar results were observed in our previous studies. Nrf2 knockdown may suppress U251MG cell growth by inhibiting cell proliferation, thus increasing cell apoptosis and inhibiting angiogenesis in mouse xenograft models^19^,^27^. In our current study, cell migration and tube information assay were performed between Nrf2 WT and Nrf2−/− BMECs. The results verified the importance of Nrf2 as a factor in angiogenesis: Nrf2 may promote migration and tube formation of BMECs involved VEGF upregulation. The consolidated results suggest that Nrf2 can regulate angiogenesis via HO-1-mediated HIF-1α/VEGF signaling pathways. In addition, certain investigations^25^,^28^,^29^ indeed indicate that HO-1 knockdown by HO-1-specific shRNA or HO-1 inhibitors ZnPP and tin-protoporphyrin may reduce the effects of Nrf2 on angiogenesis. However, the effects of Nrf2 on the pathogenesis of cerebral AVM, particularly under venous hypertension, have yet to be ecaluated mechanistically. Our findings demonstrated that the interplay between VEGF and Nrf2 regulates angiogenesis induced by intracranial VH in cerebral AVMs.

The importance of VH in the pathogenesis of AVMs is valuable in emerging studies. After Lawton *et al*. found the significant positive correlation between venous hypertension and angiogenic activity in cerebral AVMs, further studies showed that VH is an important and primary event in the pathogenesis of AVMs^5^,^6^. In 2006, Zhu *et al*. indicated that the level of angiogenic factor HIF-1α and downstream VEGF in the brain can be increased by nonischemic VH^7^. In 2009, Gao *et al*. demonstrated mild nonischemic VH results in a pro-angiogenic stage in the brain by upregulating HIF-1α and its downstream targets VEGF^30^. Similar results were observed in an experimental rat model of nonischemic VH induced by carotid–jugular anastomosis, sagittal sinus thrombosis, and transverse sinus outflow occlusion, which increased the expression of HIF-1 and its downstream VEGF^7^. In the current study, we established an experimental rat model of unilateral carotid–jugular anastomosis without sagittal sinus thrombosis, which did not only upregulate the expression of HIF-1α and VEGF but also significantly activated the Nrf2-ARE signaling pathway. To elaborate on the role of Nrf2 in the upregulation of VEGF by VH, short interfering RNA transfection *in vivo* was performed to knock down Nrf2 prior to surgery of CCA–EJV anastomosis. The results revealed that the upregulation of VEGF induced by VH was significantly inhibited by Nrf2 knockdown. In addition, our study is the first to detect the protein expression of Nrf2 and VEGF, as well as the correlation between MVDs in human AVM tissues. These findings further suggest the potential interplay between VEGF and Nrf2 in the pathogenesis of AVMs. A series of molecular signals in endothelial cells was activated by mechanical stimulation caused by hemodynamics changes without ischemia, of which VEGF is one of the most pivotal factors that stimulates angiogenesis. VEGF increased vascular permeability, resulting in the accumulation of extracellular matrix proteins, such as fibrinogen and vitronectin. These proteins induced the proliferation and migration of endothelial cells and promoted the formation of new blood vessels.

Kweider *et al*. were the first to suggest that VEGF can activate the Nrf2-ARE pathway^21^. VEGF_165_ activated the Nrf2-ARE signal pathway in the cytotrophic cell line BeWo, and this effect can be completely counteracted by the Nrf2-specific shRNA. VEGF activated Nrf2-ARE signal pathway in an ERK1/2-depentent manner. The reason is that the administration of the ERK1/2 inhibitor PD98059 can reduce the effect of VEGF-mediated upregulation of Nrf2. In addition to VEGF_165_, the Nrf2 inducer sulforaphane may increase the expression of VEGF in BeWo cells, which can also be abolished by the Nrf2-specific shRNA. With the above taken into consideration, Kweider *et al*. hypothesized that a positive feedback loop of VEGF mediated upregulation of itself via Nrf2/HO-1/CO signal pathway to promote the growth of abnormal vessels in BeWo, which may significantly influence the angiogenesis of tumors. However, whether the VEGF-Nrf2 loop exists in other diseases, particularly in cerebral AVMs, has yet to be mechanistically determined. In our *in vitro* study, primary BMECs were incubated with VEGF_165_, and the Nrf2-ARE system was evaluated by Western blot analysis. The results indicated that VEGF may activate the Nrf2-ARE signal in a dose- and time-dependent manner via the ERK1/2 signaling pathway. BMECs were then treated with the Nrf2 activator t-BHQ. The HIF-1α/VEGF signal was also markedly upregulated, which could be diminished by HO-1-specific siRNA, as determined by Western blot analysis and qRT–PCR. To verify the role of the interplay between Nrf2 and VEGF in angiogenesis, cell migration and tube formation assay were performed in both Nrf2 WT and Nrf2−/− BMECs with or without VEGF_165_ (50 ng/mL). The results further showed the crucial importance of the interplay between VEGF and Nrf2 in the pro-angiogenic effect on BMECs. All these results suggested the existence of the VEGF-Nrf2 loop in BMECs. However, the detailed mechanism of Nrf2 and VEGF regulation under VH *in vitro* needs further investigation to understand this phenomenon in BMECs.

**Figure 6:**
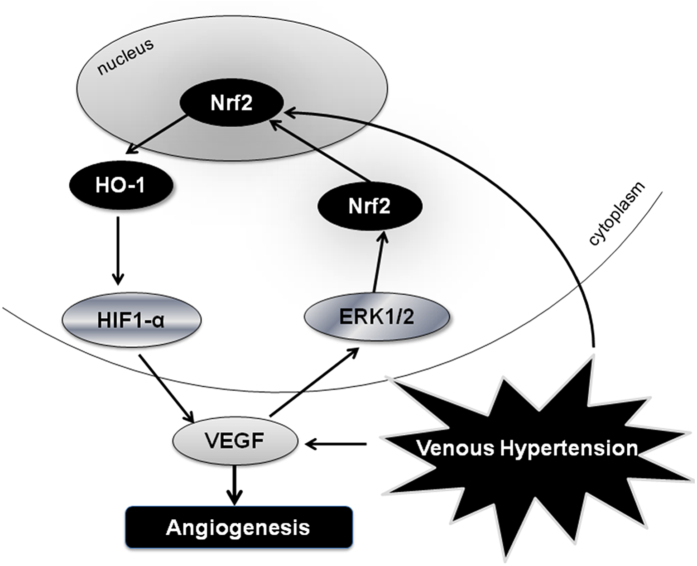
The proposed signaling pathway of this study: VH may participate in angiogenesis via activating Nrf2-ARE and HIF-1α/VEGF signaling pathway in AVMs. *In vitro*, we observed that there existed a VEGF-Nrf2 positive feed back loop in mice BMECs. VEGF activated Nrf2-ARE system via ERK1/2 signaling pathway, in turn, upregulated the expression of itself. Besides, Nrf2 palys an crucial role in the process of angiogenesis and the activator of Nrf2 t-BHQ may also upregulate VEGF expression via Nrf2/HO-1/HIF-1α pathways. Furthermore, knockout of Nrf2 impairs VEGF-induced BMECs migration and angiogenesis *ex vivo*.

As shown in Fig. 6, the salient finding of our study includes the demonstration of the involvement of the Nrf2-ARE and HIF-1α/VEGF signals induced by VH in the pathogenesis of AVMs and the existence of a VEGF-Nrf2 loop in BMECs, which may partly explain the genesis and development of AVMs. Further research has to be conducted in order to investigate the activation of the VEGF-Nrf2 loop in the BMECs stimulated by high pressure *in vitro* to mimic conditions of VH.

## Materials and Methods

### Ethics

All methods listed in the study were carried out in accordance with the approved guidelines by Research Ethics Committee of Jinling Hospital, School of Medicine, Nanjing University, People’s Republic of China.

### Patients and tissue samples

A series of 6 AVMs patents admitted at Jinling Hospital from 2014 to 2015, according to the ethical and legal standards, were included in our study. There were no gender or ethnicity restrictions on recruitment. Human normal brain tissues from the cortex were obtained from 6 patients in the pathway during surgical removal of deep benign tumor from 2015 to 2016 in Jinling Hospital. Our study was approved by the Research Ethics Committee of Jinling Hospital, School of Medicine, Nanjing University, People’s Republic of China. Written informed consent was obtained from all the patients included in our study.

### Animals

Male Sprague-Dawley rats and newborn ICR mice, including Nrf2 knockout mice were used for this study. The experimental protocols in the present study including all the surgical procedures and animal usages were in line with the Guide for the Care and Use of Laboratory Animals by the National Institutes of Health (NIH) and approved by the Animal Care and Use Committee of Nanjing University. Nrf2 knockout ICR mice were kindly provided by Dr. Thomas W. Kensler (Johns Hopkins University, Baltimore, MD, USA).

### RNA Transfection

We performed *in vivo* short interfering RNA (siRNA) transfection targeting Nrf2 according to the method described previously^31^. The stereotaxic coordinates were 1.3 mm posterior, 5.0 mm lateral to the bregma, and 4.5 mm ventral to the surface of the skull. Then, 5 μL Nrf2 siRNA (Genechem, Shanghai, China) or 5 μL scramble (Genechem, Shanghai, China) diluted with the same volume of transfection reagent (Engreen Biosystem Co, Ltd. China) was mixed gently and incubated 45 min at room temperature. Finally the mixture was injected intraparenchymally using a microsyringe under a guidance of stereotaxy instrument. After 24 h of transfection, animals was used for the following experiment procedures to establish animal models.

*In vitro*, synthetic small interfering RNA (siRNA) targeting HO-1 was transfected using Lipofectamine™ 2000 (Genechem, Shanghai, China) to knock down the expression of HO-1 transcripts in BMECs. Control siRNAs encoded scrambled sequences that should not lead to the specific degradation of HO-1 mRNA. BMECs were transduced with siRNA-HO-1, siRNA-control, respectively, for 48 h before being used in experiments.

### VH Model in the Rat

To exclude any potential hormonal effects on VH formation^32^, only male Sprague–Dawley rats weighed 350–400 g were used in our study. Sixty rats were randomly assigned to the following subgroups: sham surgery of Nrf2 WT rats (n = 12), sham sugery of Nrf2 scramble rats (n = 6), sham surgery Nrf2i rats (n = 6), CCA–EJV anastomosis of Nrf2 WT rats (n = 24), anastomosis treated with scramble (n = 6), and anastomosis treated with Nrf2 siRNA (n = 6). All animals were fasted for 12 h before surgery without water restriction. A rat VH model was performed modifying the method described previously^33^. The rats were anesthetized by the intraperitoneal anesthesia with pentobarbital sodium (50 mg/kg) and the anesthetic was supplemented as needed during the surgical procedures. All the surgical procedures were performed using standard sterile techniques. After anesthesia, the rat was fixed on the operating table. Following a ventral midline neck incision, the left common carotid artery and the left external jugular vein were exposed and placed side by side. Then, temporary clips were used to the proximal and distal portions and a side-to-side anastomosis was carried out using no. 10-0 monofilament nylon suture under a microscope. Afterwards, the proximal portion of the left external jugular vein was occluded. The wound was irrigated with sterile saline and the incision was sutured with no. 3-0 monofilament nylon. By comparison, a similar surgical procedure without creating left common carotid artery–external jugular vein anastomosis was performed as a control model. The CCA–EJV anastomosis of Nrf2+/+ rats were euthanized on days 1, 3 and 7 after sugery. The rats of anastomosis treated with scramble, and Nrf2 siRNA were killed on day 1. (n = 6 per group).

### Primary Culture and Identification of BMECs

Primary culture brain microvascular endothelial cells were performed with some modifications on the methods described previously^34^. Brains were obtained from postnatal 3 to 5 days Nrf2−/− and Nrf2 WT mice (6 mice for each genotype) and stored in DMEM/F12 on ice. And surface meninges, cerebellum and brain stem were removed using a sterile Ophthalmic forceps. The isolated mice brains were minced with a ophthalmic scissors, digested in at 37 °C with 0.7 mg/mL type 2 collagenase and 39 U/mL Dnase I in DMEM/F12 for 1.25 h, and agitated every ten minutes. Then the enzyme solution was diluted with DMEM/F12 and centrifuged at 1000 g for 5 min at 4 °C. The pellet was re-suspended in a 20% w/v bovine serum albumin and DMEM/F12 solution and centrifuged for 20 min at 4000 g to obtain a microvessel enriched cell pellet. Afterwards, the microvessel suspension was dissociated using 0.3 mg/mL collagenase/dispase and 11 U/mL Dnase I in DMEM/F12 at 37 °C for 1 h. The digested microvessel solution was diluted with DMEM/F12 and centrifuged at 1000 g for 5 min. Subsequently, the pellet was washed twice with DMEM/F12, re-suspended in complete culture medium and seeded in polylysine- coated 60-mm cell culture dishes incubated at 37 °C with 5% CO2 in air. Culture medium consisted of DMEM/F12 supplemented with 20% FBS, 1% Endothelial Cell Growth Supplement (ECGS, ScienCell), 100 U/mL penicillin and 100 μg/mL streptomycin. The culture solution was changed in the next 36 h to pury the cells. The endothelial phenotype was confirmed by immunocytochemistry analysis for VIII factor (sc-14014, Santa Cruz Biotechnology, CA, USA) (see Supplementary Fig. S1).

### Immunohistochemistry

At 1, 3, 7 d after the surgery, rats were anesthetized and perfused with 4% buffered paraformaldehyde respectively. The brain were carefully removed out and stored in 4% buffered paraformaldehyde. The sections were embedded in paraffin and cut into 5-μm thickness. For immunohistochemical staining of Nrf2, the sections were deparaffinized in xylene and rehydrated. Endogenous peroxidase was blocked with 3% H2O2 in methanol at room temperature for 15 minutes. The slides were rinsed with phosphate-buffered saline and incubated with 0.3% bovine serum albumin in phosphate- buffered saline for 30 minutes at room temperature. Primary antibodies against Nrf2 (1: 100; Abcam, Cambridge, MA, USA) were applied overnight at 4 °C. After being washed three times in PBS for 5 min each, the sections were incubated with horseradish peroxidase-conjugated IgG (1:500; Santa Cruz Biotechnology) for 60 min. 3,3-Diaminobenzidine (DAB)/H2O2 solution was used to visualize Nrf2. Before the sections were mounted, cell nuclei were counterstained with hematoxylin. The intensity of immunohistochemical staining was performed with quantitative image analysis.

### Western Blotting

To prepare total protein lysates, cells were harvested and lysed in cold RIPA buffer (1% NP40, 0.5% sodium deoxycholate, 0.1% SDS, 1 mM EDTA, 1 mM EGTA, 1 mM Na3VO4, 20 mM NaF, 0.5 mM DTT, 1 mM PMSF, and protease inhibitor cocktail in PBS pH 7.4) and centrifuged at 13,000 g for 5 min at 4 °C to remove debris. (To prepare the cytoplasmic and nuclear protein, cells were lysed using a nuclear and cytoplasmic protein kit (Beyotime) according to the manufacturer’s instructions. Protein concentrations were estimated by Coomassie Plus Protein Assay Reagent (Pierce, IL, USA). Fifty micrograms of the resulting cytosolic protein extracts were heat denatured in Laemmli sample loading buffer, separated by 8–15% sodium dodecyl sulfate polyacrylamide gel electrophoresis using the Criterion system (Bio-Rad, Hercules, CA) and transferred to polyvinylidene fluoride (PVDF) membranes. After blocking with 5% non-fat dried milk for 2 h at room temperature, the membrane was incubated with the primary antibodies overnight at 4 °C. The following antibodies were used: anti-β-actin (sc-130657, Santa Cruz Biotechnology, 43 kDa), anti-Nrf2 (sc-722, Santa Cruz Biotechnology, 68 kDa), anti-HO-1 (sc-10789, Santa Cruz Biotechnology, 32 kDa), anti-NQO1 (ab34173, Abcam, 31 kDa), Anti-VEGF (ab46154, Abcam, 44 kDa), and anti-HIF-1α (ab51608, Abcam, 110 kDa), anti-ERK1/2 (137F5, Cell Signaling Technology, 42/44 kDa) and anti-p-ERK1/2 (197G2, Cell Signaling Technology, 42/44 kDa). Each primary antibody was diluted appropriately in blocking buffer and then icubated overnight at 4 °C. The blots were washed three times in the washing buffer and incubated with the horseradish peroxidase (HRP)-linked secondary antibody (Cell Signaling, Danvers, MA; 1:2000) at room temperature for 2 h. After washing, blots were incubated with enhanced chemiluminescence (ECL) detection system (Amersham Biosciences, Bucks, UK) and exposed to X-ray film (Fuji Hyperfilm, Tokyo, Japan). Quantity One softerware 4.6.2 (BioRad) was used to analyze the mean pixel density of the blots. β-actin was used as a housekeeping gene.

### Quantitative Real-Time PCR (q-RT PCR)

Q-RT PCR was performed following the previous method^35^. Primer sequences using in our study were as follows: Nrf2 forward and reverse primers were 5′-TGAAGCTCAGCTCGCATTGA-3′ and 5′-TGCTCCAGCTCGACAATGTT-3′; HO-1 forward and reverse primers were 5′-ATCGTGCTCGCATGAACACT-3′ and 5′-CCAACACTGCATTTACATGGC-3′; NQO1 forward and reverse primers were 5′-CATTCTGAAAGGCTGGTTTGA-3′ and 5′-CTAGCTTTGATCTGGTTGTCAG-3′; VEGF forward and reverse primers were 5′-GAAGGAGGAGGGCAGAAT-3′ and 5′-CGATTGGATGGCAGTAGC-3′; HIF-1α forward and reverse primers were 5′-AAGTCAGTGTACAGGCCAGC-3′ and 5′-CTCGGCTAGTTAGGGTACACTT-3′; β-actin forward and reverse primers were 5′-CTGAATGGCCCAGGTCTGAG-3′ and 5′-AAGTCAGTGTACAGGCCAGC-3′.

### *In vitro* angiogenesis assay

*In vitro* endothelial cell tube formation assays were carried out as previously described^36^. Briefly, BMECs were washed with PBS three times, detached with 0.05% trypsin/EDTA (Invitrogen), centrifugated with 2000 rpm for 5 min, washed with PBS again and counted with a hemocytometer. Besides, Matrigel Basement Membrane Matrix (356234, Coring) was warmed up at room temperature and transferred onto ice before completely thawed. Then, 50 μl of Matrigel was plated to 96-well plates at a horizontal level, and incubated for 30 min at 37 °C. Afterwards, BMECs (2 × 10^4^) of Nrf2−/− and Nrf2 WT were re-suspended with serum-free DMEM/F12 with or without VEGF_165_ (100 ng/ml) and loaded on the top of the Matrigel. Each group contained 6 wells. Following incubation at 37 °C overnight, each well was analyzed directly in five distinct low-power fields under an inverted phase contrast microscope using ImagePro Plus (verson 6.0). Each experiment was repeated three times, and each treatment was probed in 6 wells.

### Cell migration assay

BMECs migration was determined using a trans-well chamber (8.0 μm membrane pores, Corning, USA) assay. Briefly, sersum-starved cells were trypsin-harvested in FBS-free DMEM/F12. Next, 500 μl of DMEM/F12 containing 1% FBS with or without VEGF_165_ (100 ng/ml) was added to the lower chambers, while BMECs (2 × 10^4^) of Nrf2−/− or Nrf2 WT were plated in the upper chamber. After 12 h of incubation, the cells on the top surface of the membranes were removed by cotton swabs and the cells on the bottom surface were fixed with 4% paraformaldehyde at 37 °C for 20 min and stained with Gentian violet at 37 °C for 10 min. Migrated cells were counted under a Zeiss phase contrast microscope in five random high-power fields. All groups of experiments were performed in triplicate.

### Statistical analysis

SPSS 20.0 software (SPSS, Chicago, IL) was used for statistical analysis. All results were expressed as mean ± SEM. One-way analysis of variance (ANOVA) followed by Tukey post hoc comparison tests was used to compare the levels of different experimental groups. p-Values of <0.05 and <0.01 were considered statistically significant and very significant, respectively.

## Additional Information

**How to cite this article**: Li, L. *et al*. Interplay between VEGF and Nrf2 regulates angiogenesis due to intracranial venous hypertension. *Sci. Rep*. **6**, 37338; doi: 10.1038/srep37338 (2016).

**Publisher’s note:** Springer Nature remains neutral with regard to jurisdictional claims in published maps and institutional affiliations.

## Supplementary Material

Supplementary Information
