# Supplementary material for: Interplay between VEGF and Nrf2 regulates angiogenesis due to intracranial venous hypertension
Source: Sci Rep. 2016 Nov 21;6:37338. doi: 10.1038/srep37338 (PMC5116754; doi:10.1038/srep37338)
Supplement: Supplementary Information [file srep37338-s1.pdf]

Supplementary Information for:

Interplay between VEGF and Nrf2 regulates angiogenesis due to intracranial venous hypertension

Liwen Li[1], Hao Pan[1*], Handong Wang[1**], Xiang Li[1], Xiaomin Bu[2], Qiang Wang[1], Yongyue Gao[1], Guodao Wen[1], Yali Zhou[1], Zixiang Cong[1], Youqing, Yang[1], Chao Tang[1],Zhengwei, Liu[1]

[1]Department of Neurosurgery, Jinling Hospital, School of Medicine, Nanjing University, Nanjing, China

[2]Department of Clinical Laboratory, Jinling Hospital, School of Medicine, Nanjing University, Nanjing, China

*Correspondence to: Hao Pan, Department of Neurosurgery, Jinling Hospital, School of Medicine, Nanjing University, 305 East Zhongshan Road, Nanjing, 210002, China. E-mail address: panhao_nz@163.com

**Co-Correspondence to: Handong Wang, Department of Neurosurgery, Jinling Hospital, School of Medicine, Nanjing University, 305 East Zhongshan Road, Nanjing, 210002, China. E-mail address: njhdwang@hotmail.com

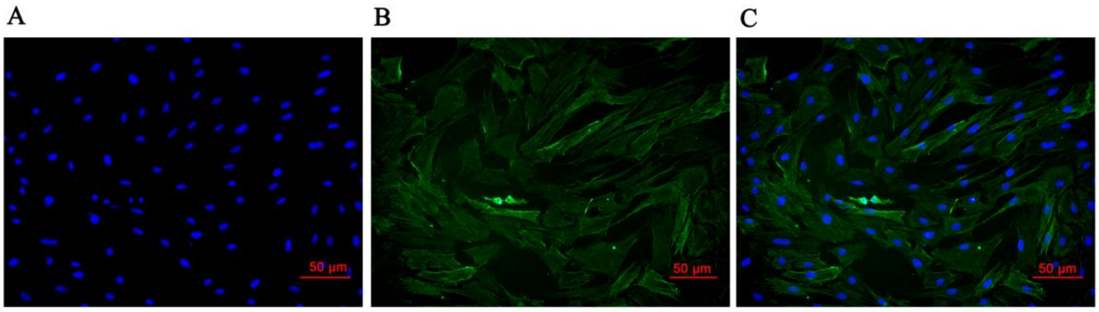

Supplementary Figure S1. Immunofluorescent labeling of factor VIII

Fig. S1.A was DAPI stained image showing nuclei (blue), Fig. S1.B showed factor VIII staining (green), Fig. S1C showed the merged image of these two images. Immunofluorescence stain for factor VIII showed typical microvascular elongated morphology and formed confluent layers, which indicated that majority of cells were BMECs. Bar:50 μm.
